# Indents

**Published:** 1907-10-15

**Authors:** 


### INDENTS.

☞Brief Articles of Techinical or Scientific Value will receive notice and comment. Contributions invited.

**The Treatment of Pyorrhea Alveolaris by Opsonins.**

This issue of *The Dental Brief* contains a valuable paper on the treatment of pyorrhea alveolaris by the opsonic method, as practiced by Dr. G. B. Webb, of Colorado Springs, Colo. Dr. Webb was the former assistant of Drs. Wright and Douglas, the noted bacteriologists, of London, who have secured wide reputation through the treatment of typhoid, tuberculous and other diseases by bacterial vaccines. This method in pyorrhea alveolaris is not to be looked upon as a "cure-all," applicable to every case. All calcic deposits must, of course, first be removed by surgical manipulation, and the vaccine resorted to only as an after or preventative treatment, cultures being made for each individual case. Dr. Webb contends that if this is carefully done a cure will be effected. "Stock vaccine," which doubtless will eventually be manufactured and put on the market by drug supply houses, will, however. not accomplish the work, it being necessary, as above stated, that individual cultures shall be made for each case. The opsonic method of treatment for pyorrhea alveolaris seems to possess merit, and to be worthy of investigation and experimentation by the dental profession. For the medical profession numerous and valuable papers have been written on this subject. What is probably one of the best will shortly appear in *The Journal of the American Medical Association*; it is from the pen of Dr. Leary, of Boston, and was read at the recent meeting of the Medical Association at Atlantic City.—Dr. Thorpe, in *Brief*.

**Baking Porcelain.—C. H. Land, Detroit.**

A Voice—How do you support that porcelain when in the furnace?

Dr. Land—I get a very thin piece of platinum and lay it on top of a fire-brick slab, and sometimes I get a little trough and lay it on that to keep it off the fire-brick and clean. If there is anything in this world you want to keep clean and keep dirt and flux away from, it is porcelain. I don't know anything so sensitive to dirt as porcelain. You must watch it closely and rest it on a clean surface. If you put it on dirt you will find a hole in it, and if you grind it you will get a piece of carborundum in it. I will tell you how to get rid of the carborundum. Never put a tooth, a vitrified piece of porcelain, back in the furnace after you have ground it, until you have washed it and fired it again, because if you do, there will be a little bit of dirt, and you will have three or four nasty little bubbles on the front of the tooth. If you besmear a plate-glass window you cannot see through it, because light is refracted. A large amount of flux put in porcelain makes it opaque and not translucent. The less flux you have in a body the more translucent it will be. A true dental porcelain is a porcelain that will hold its form both before and after fusing; that is, I want a porcelain I can just mix with water and build up in my hand, and then put it through the fire and not get it out of shape. I have been asked how long I leave my porcelain to cool. Probably a high-grade porcelain, unless it is quite a large molar, can be taken out and thrown all over the room, barring getting dirty, and would never crack. A low-grade will crack as soon as you take it out and cool it. I have made crowns out of pure silica under the oxyhydrogen blowpipe, and heated them red-hot and thrown them in the water, and you cannot crack them. With porcelain, if you could reach the fusing point of silica, you could throw it in water. To devitrify most of our porcelains, we can throw them in water, but when the flux is all out and it is reduced to pure silica, you can throw it in water and it does not break. All of our clays are silicates of aluminum, and the less flux there is in them the less liable they are to crack. If you want to be careful with the porcelain that is easy to fracture or crack, put it in the muffle on a comparatively thick piece of fire-brick. Take it out in the open air, leave it on the brick and let it cool, and it will never crack, because the brick is a non-conductor and holds the heat long enough to temper any ordinary piece. Do not lay it on anything that will absorb the heat from it. A substance generally throws away heat about as fast as it takes it. Different metals in different clays will do the same thing. They receive and give away about the same proportion. So all of our fire-brick, fire-clays, are slow to give up heat, and with small pieces like that there is no danger whatever, of cracking if you take them out immediately. —*Dominion Dental Journal.*

**Seamless Crowns.** While there are many and varied methods of detail of construction of seamless crowns, there is but one general line of procedure which will give results which are sufficiently reliable to insure an approach to the necessary degree of accuracy. This procedure consists of taking the measurement of the prepared root and fitting a primary band, the exact shape and conformation of which is subsequently reproduced in the finished crown. The primary band may be made, preferably, of 30-gauge copper. It might be well to say just here that after having made the few seamless crowns the supply of bands will be quite sufficient and you will seldom have to take the time to make new bands, as it is an easy matter to save them. Select a punch that will fit the wire measurement, and you will find this to be the correct size of the copper band. Place the band in position on the prepared tooth. Then with Perfection modeling composition take the bite with the band in position. When the impression material is sufficiently chilled remove the band and place it into the impression; pour each side, articulate and separate the bite. The occluding surfaces of the opposing teeth are varnished, the stump is built up with fresh plaster and the bite is closed. Then when the plaster has become sufficiently hard we can carve the crown to the desired shape. Cut away a little plaster underneath the band and separate the adjoining teeth with a thin ribbon saw; remove the carved tooth from the model. Then when the tooth is sufficiently dried place it in mouldine, prepare the ring and pour. When the model has become hard, knock it out of the ring, remove the cardboards and with a chisel or knife split the metal and remove the plaster tooth. Inclose the shell into the space formerly occupied by the plaster tooth, place it into the iron ring and now begin the swaging. We have obtained the best results in swaging by using a soft pine stick in the early part of the operation. Anneal, replace in the metal die and give the final swaging with gutta percha and a hardwood stick, being careful to shape this stick somewhat like the shape of the crown. After this has been done, if the proper precautions have been taken, the crown should be completed so far as swaging is concerned. It is now necessary to trim away the surplus gold around the neck of the crown and polish it down to the required thinness. The solder should not be flown into the cusps until after the crown has been tried and fitted in position; then it requires but a few moments for the operation of soldering and polishing.

If the crown to be swaged is decidedly bell-shaped, and the cusps prominent, we may have a bursting or tearing of the gold. This is easily mended by placing a little pellet of gold foil in the rent and packing the foil down with some gum camphor; then hold the crown over the flame until the camphor has burned away; by dropping a piece of solder upon the pellet and again holding the crown over the flame the trouble is easily corrected.—E. E. Tydings, *Dental Era.*

**Gold Inlays: An Original Method.** This inlay takes the place of the large contour gold filling we are wont to put in the anterior teeth. It requires about half the time. The rubber dam need not be used, and, unlike the gold filling we spend hours to put in, cannot possibly be bitten out. There is no danger of pitting or peeling, and the inlay is susceptible of a much higher polish.

Prepare the tooth as you would for a large gold filling, by removing the pulp and filling the canal. Prepare the cavity without under cuts, leaving it saucer-shaped, concave-labio—lingually. This gives direct access to the root canal. With a plug-finishing burr and Arkansas stone, leave the margins sharp. By drilling in a piece of wood, find the diagonal of the square post to be used. Use a square post, because its four points of contact will assist you very materially in holding the base of the matrix while burnishing.

Now roughly burnish a piece of 40-gauge platinum plate to the floor and walls of the cavity (if it may be said to have walls), insert the pin, remove and tack with pure gold. Replace and burnish again to get the outline of the margins. Cut away the bulk of the surplus, burnish again, and if the platinum has a tendency to spring up at the cutting edge, flow pure gold over the inner surface, then with a suitable gold plugger mallet every portion of the base to contact. Cut off the platinum pin you have been using to handle the work with and trim away the surplus plate flush with the margins.

On this base make and contour the gold filling, so to speak, out of wax or Parrs flux exactly as you want it to be when finished. Keep the wax flush with the margins of the tooth. Now cut a piece of inlay platinum foil large enough to wrap around the wax, smoothly covering the labial surface; bring the foil down over the cutting edge. Get the mesial or distal contour, and leave the remaining lingual surface open. With a hot instrument touch the foil gently. This will cause it to stick so that it may be handled. Remove and paint the pin and platinum plate with whiting or rouge to prevent the gold from flowing where it is not wanted, and invest. Pick, melt or wash out the wax and solder with pure gold.

Old gold fillings may be utilized, but should be melted into globules first. The labial surface may be pure gold and the remainder any karat your scraps afford. Again, the cutting edge can be made 22-karat and the remainder pure gold. Almost any color and degree of hardness may be obtained. After soldering cool in water and cement to place. When the cement is sufficiently hard, finish as you would a gold filling. The platinum foil is removed with the first strip.

One would naturally think that the platinum plate along the margin would show, but it does not, owing to its becoming so thoroughly impregnated with the gold under the blow pipe.—D. D. Campbell, in *Brief*.

**Bicuspids and Molars.** With these teeth, the Ivory matrix should be adjusted, and an impression taken with Perfection compound, by taking a small piece in the form of a cone. Heat only the small end, and force it into the cavity; hold it firmly and chill with ice water. After it is thoroughly chilled, it can be removed unless you have overlooked some undercuts which should have been filled previously with cement.

This impression is then taken to the laboratory and a thin film of vaseline (the thinner the better) is applied. The next step is to take a good cement and mix to the consistency of stiff biscuit dough, and by holding the impression between the fingers of one hand, the cement may be forced into all of its inequalities; and when thoroughly set—usually taking twenty to thirty minutes—can be separated, and the exact reproduction of the cavity is the result. This cement model is then placed in a small swedger, and over the cavity a small piece of pure gold .003 inch in thickness, simply laid over the model. Now adjust your swedger, and with one blow of the sledge your matrix is made. Should a hole be torn in the matrix, press over this a small piece of moss fibre gold and reswedge. With sticky wax flow your matrix full, and carve approximately to the lost portion of the tooth

Chill with ice water and remove from model. If you wish to be accurate, and avoid much unnecessary grinding, place this in the cavity, have the patient close his jaws; in this way you get the exact occlusion. Always chill your wax with ice water before removing.

To make an inlay for a contour of a central incisor. The matrix for impression in this case is obtained by placing a metal strip between the teeth and taking the compound as before, force it into the cavity, holding it firmly with one hand, and with wet fingers, and the thumb of the other, press it from the palatal and labial aspect, until the material becomes hard. You may now remove your fingers and apply ice water. The model and matrix are obtained in the same way as in molars and bicuspids.

The matrix is now made, and the lost portion of the tooth has been restored in this matrix. The next step would be to cast your inlay. Take a small amount of Brophy's Imperial Compound, mix to moderate stiffness, and first apply to the cavity side of the matrix; being careful to work out all air spaces. Then invest wax and all, having a small, funnel-shape opening, usually on approximal side of bicuspids and molars, and palatal side of incisors and cuspids, through which the wax is flushed out with boiling water. Now take small gold shot (the smaller the better) of 22K plate, and fill your funnle full; place in furnace and turn on your heat. When it is properly fused you will note the settling together of these little globules. At this time, with long-handle pliers, remove from the furnace, at the same time giving it a slight jolt. If you fail to have gold enough add more and refuse.

Never use solder or flux in this work. Solder contracts too much and flux causes your matrix to fuse.—J. L. Bridgeford, in *Western Journal.*

**The Lower Plate.** Doubtless many have their own methods of securing atmospheric pressure on full lower plates, and some may never have tried it at all; so, in the hope of bringing out a discussion on the subject,

I shall endeavor to describe a method which I have used for several years for securing atmospheric pressure on the full lower plate. For impression material I have used modeling compound—Perfection compound has given as good satisfaction as any. Plaster of paris, of course, is an ideal impression material, but it does not give the kind of impression that is desired in this method.

The compound is warmed to a consistence at which it will adapt itself readily to the shape of the mouth, place upon the tray, put in the mouth, and pressed down almost as far as you desire it to go. Then wait for a minute or two until it hardens slightly. During this interval of time I have my assistant direct a current of cold air into the mouth from a rotary fan in the dental engine, which procedure chills the outer layer of the compound and prevents it from flowing away from the jaw when pressure is put upon it. While the layer of impression material that is in contact with the tissues is still warm and plastic, the tray is pressed down a little farther and held steadily—with about half the force used in pressing it down the first time—until it is quite hard. The tray is then removed and the cast is poured.

In order that we may know why we proceed in this manner, let us go over the taking of this impression again. In the first place, when the material is put in the mouth and pressed down we get an ordinary impression; after it is allowed to stand a little while and the outside is chilled and pressed down a second time, the impression is changed from the ordinary one, but in what way? When pressing down on the tray the second time, the hard ridge of the alveolus cuts just a little deeper into the impression material, and remains practically unchanged, while the soft tissues down on the sides of the gums, about where the border of the plate comes, are compressed slightly. When the plate is made on the model from this impression and put into the mouth, the margins of the plate are sealed all the way round, and this converts the whole lower surface of the plate into one great suction-pad—if we may use that term.

One of the most delicate parts of the operation is the trimming of the plate to just that line that will best seal its borders, for if it be too long it will give the muscles an opportunity to displace it; if too short it may allow the air to pass in, and thus defeat our whole purpose.

It is self-evident that the upper plate is retained by air-pressure against gravity, while the lower plate adds its own weight to atmospheric pressure; and so, compared with the upper, the lower has twice its own weight to add to the atmospheric pressure, thus showing that it is a better subject to be retained by such pressure than the upper plate.

The lower plate has always been a *bete noire* in dentistry. Of course we all would rather fill, treat, crown or bridge to meet the conditions we find in the mouth, but occasionally there come before us conditions for which the science and art of dentistry has nothing better to offer than a plate. How necessary, then, both for the comfort of the patient and for our own credit, that it be made to stick.—D. H. Young, in *Dental Cosmos*.

**Somnoform.** Dr. C. M. Paden, of Chicago, has made a research on the anesthetic properties of somnoform and its toxic tendencies and publishes in the September *American Journal* his conclusions in the following summary:

First, That somnoform is not, under normal conditions, a depressant. We have seen that the pulse and respiration are at first stimulated and then remain about normal unless anesthesia is pushed to the limit. There have been abundant proofs of this on both animals and patients. We have also seen that the blood-pressure in animals shows but little change during long periods of anesthesia unless outside interference occurs and that it then quickly returns to normal. As related in the account of the last experiment, the blood-pressure in man after five minutes of complete anesthesia shows remarkably little change. These results are of the greatest importance to the anesthetist. They indicate first that the respiratory centres are not depressed and that normal quantities of air are continuously inspired. When considered in connection with the rapid elimination of somnoform, this means that few patients will show signs of collapse, that it will not ordinarily be far reaching and that should resuscitation become necessary, the condition of the respiratory centres is such as to greatly assist.

The pulse and blood-pressure records are not less important. They indicate an excellent tonic condition of the centre even after long anesthesia and equally good tonic condition of the heart and blood vessels. Just as you cannot have good water pressure unless both the pumps and pipes are in good condition, so you cannot have normal blood-pressure unless this centre governing the activity of the heart is in good condition and the vaso-motor centres maintain the blood vessels in normal calibre.

A proper condition of these centres insure that the oxygen inspired by the lungs shall be properly and promptly distributed to the nerve centres and tissues and become the most important of all supporting and resuscitation forces.

In view of these results the writer feels that somnoform belongs in the same class as ether so far as its action on the blood-pressure goes. It certainly does not belong in the class with chloroform, which is a heart depressant. Its standing as regards safety must be taken from the history of its administrations. The writer is of the opinion that as regards safety it must be classed with nitrous oxide. The writer has been able to learn of four authentic deaths two in this country and two in others. The writer does not know how many administrations there have been, but he has records 72,641 by large users, with the most satisfactory results and thinks there must have been more than 500,000 since the International Dental Congress at St. Louis, Mo., 1904. Each of the anesthetists reports complete satisfaction (with the exception of one) and many added words of praise. He also chanced to meet one of the members of the faculty of the Dental Department, University Illinois, who informed him that somnoform had been administered 1,500 times in their clinics with excellent results. It therefore seems necessary to conclude that somnoform should be classed with nitrous oxide as to safety, and the records class it as much safer than ethyl chloride and ethyl bromide, from each of which a considerable list of fatalities are reported.

As regards convenience, somnoform stands alone in the the ease of administration, speed with which anesthesia is induced, the duration of anesthesia and speed and completeness of recovery.

